# The genome sequence of an anthomyiid fly, *Eustalomyia histrio *(Zetterstedt, 1838)

**DOI:** 10.12688/wellcomeopenres.21264.1

**Published:** 2024-04-24

**Authors:** Steven Falk, Philip Brighton, University of Oxford and Wytham Woods Genome Acquisition Lab, Darwin Tree of Life Barcoding collective, Wellcome Sanger Institute Tree of Life Management, Samples and Laboratory team, Wellcome Sanger Institute Scientific Operations: Sequencing Operations, Wellcome Sanger Institute Tree of Life Core Informatics team, Tree of Life Core Informatics collective, Darwin Tree of Life Consortium

**Affiliations:** 1 Independent researcher, Kenilworth, England, UK; 2 Organiser of UK Anthomyiidae Recording Scheme for the Biological Records Centre, Warrington, England, UK

**Keywords:** Eustalomyia histrio, Anthomyiid fly, genome sequence, chromosomal, Diptera

## Abstract

We present a genome assembly from an individual male *Eustalomyia histrio* (Anthomyiid fly; Arthropoda; Insecta; Diptera; Anthomyiidae). The genome sequence is 871.3 megabases in span. Most of the assembly is scaffolded into 6 chromosomal pseudomolecules. The mitochondrial genome has also been assembled and is 19.42 kilobases in length. Gene annotation of this assembly on Ensembl identified 26,785 protein coding genes.

## Species taxonomy

Eukaryota; Opisthokonta; Metazoa; Eumetazoa; Bilateria; Protostomia; Ecdysozoa; Panarthropoda; Arthropoda; Mandibulata; Pancrustacea; Hexapoda; Insecta; Dicondylia; Pterygota; Neoptera; Endopterygota; Diptera; Brachycera; Muscomorpha; Eremoneura; Cyclorrhapha; Schizophora; Calyptratae; Muscoidea; Anthomyiidae; Anthomyiinae; *Eustalomyia*; *Eustalomyia histrio* Zetterstedt, 1838 (NCBI:txid2881975).

## Background

*Eustalomyia* are typical muscoid flies in terms of shape and size, but they do have a marked pattern of whitish grey and black on the body which set them apart from most Anthomyiidae. Ackland *et al.* (2017) give a key for identification of the genus amongst the British Anthomyiidae. *E. histrio* is separated from the very similar but more common *E. festiva* by details of hairing on the antennal arista and setae on the hind tibiae. Hennig (1976) provides illustrations of the head, phallus and ovipositor while the male external genitalia are illustrated in Ackland *et al.* (2017). Hennig (1976) listed five European species in this genus, while the Global Biodiversity Information Facility states that one of these ( *lepraota* Séguy) is now regarded as synonymous with *vittipes* Zetterstedt ( GBIF Secretariat, 2024).

The most closely related genus of Anthomyiids according to Hennig (1976) is *Leucophora*, which are also cleptoparasites of aculeate Hymenoptera. This close relation has been corroborated by a molecular phylogenetic study ( Li *et al.*, 2023).

In Britain *Eustalomyia histrio* is an uncommon but not scarce species, distributed throughout England and Wales from the South coast to Lancashire and Yorkshire ( NBN Atlas Partnership, 2023). More than half the records of *E. histrio* are from the months of May and June, falling off in July to a low level in August and September ( NBN Atlas Partnership, 2023). There are a very few outlying records from Scotland ( Horsfield, 2002), but it has not been recorded from Ireland ( Chandler, 2023). There are numerous records on GBIF of this species in the northern parts of Europe and Scandinavia ( GBIF Secretariat, 2024). Huckett (1971) gives a list of North American locations extending from California to Nova Scotia. In addition, Suwa (1999) records the species from Japan, Korea and Kamchatka.

Flies of the genus *Eustalomyia* are recorded as cleptoparasites of solitary wasps in the family Crabronidae nesting in rotten wood ( Bland, 1999; Falk & Pont, 2017; Hennig, 1976; O’Toole, 2010). The adults are generally found in the vicinity of wood likely to harbour such nests, but their habits are otherwise unknown. They possibly feed on nectar or pollen like many other Anthomyiidae ( Falk, 2021). O’Toole (2010) lists only the wasp genus *Crabro* as a host, while other *Eustalomyia* have been recorded in association with various *Crossocerus*, *Ectemnius* and *Trypoxylon* species. There is insufficient information to establish whether this represents a definite specialisation.

Apart from Bland’s (1999) description of the rearing of larvae, there are no detailed studies of the effects of *Eustalomyia* parasitism on host species. This is a lifestyle confined to rather few Diptera genera from widely separated families ( O’Toole, 2010). How the few *Eustalomyia* species can be both so widespread, across at least three continents, and with such an apparently specialised niche requirement is an interesting evolutionary and biogeographical question. Genomic studies such as the present one may shed light on this.

## Genome sequence report

**Figure 1.**
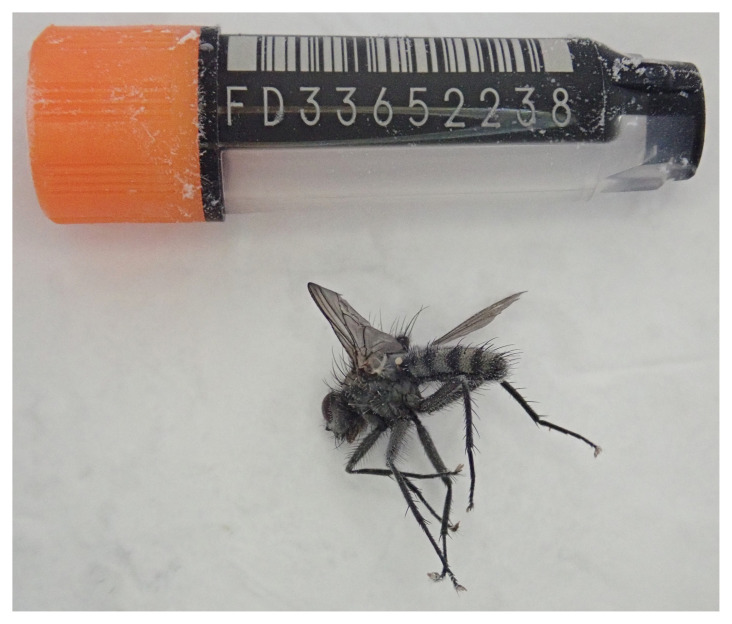
Photograph of the *Eustalomyia histrio* (idEusHist1) specimen used for genome sequencing.

The genome was sequenced from one male *Eustalomyia histrio* ( Figure 1) collected from Wytham Woods, Oxfordshire, UK (51.77, –1.33). A total of 24-fold coverage in Pacific Biosciences single-molecule HiFi long reads was generated. Primary assembly contigs were scaffolded with chromosome conformation Hi-C data. Manual assembly curation corrected 99 missing joins or mis-joins and removed 18 haplotypic duplications, reducing the assembly length by 0.90% and the scaffold number by 28.57%, and decreasing the scaffold N50 by 77.19%.

**Figure 2.**
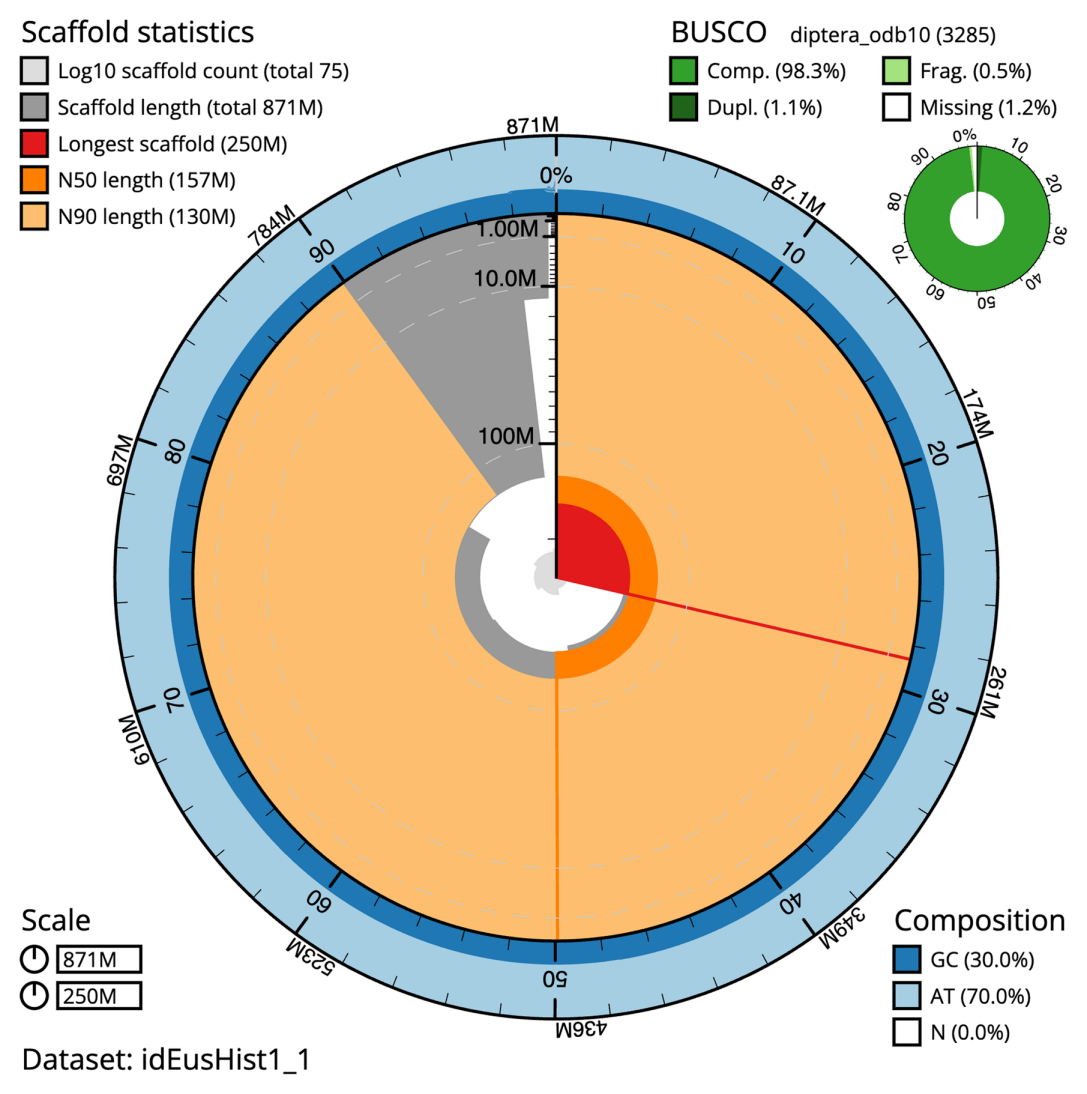
Genome assembly of *Eustalomyia histrio*, idEusHist1.1: metrics. The BlobToolKit snail plot shows N50 metrics and BUSCO gene completeness. The main plot is divided into 1,000 size-ordered bins around the circumference with each bin representing 0.1% of the 871,342,778 bp assembly. The distribution of sequence lengths is shown in dark grey with the plot radius scaled to the longest sequence present in the assembly (249,786,838 bp, shown in red). Orange and pale-orange arcs show the N50 and N90 sequence lengths (156,857,283 and 129,770,906 bp), respectively. The pale grey spiral shows the cumulative sequence count on a log scale with white scale lines showing successive orders of magnitude. The blue and pale-blue area around the outside of the plot shows the distribution of GC, AT and N percentages in the same bins as the inner plot. A summary of complete, fragmented, duplicated and missing BUSCO genes in the diptera_odb10 set is shown in the top right. An interactive version of this figure is available at https://blobtoolkit.genomehubs.org/view/idEusHist1_1/dataset/idEusHist1_1/snail.

**Figure 3.**
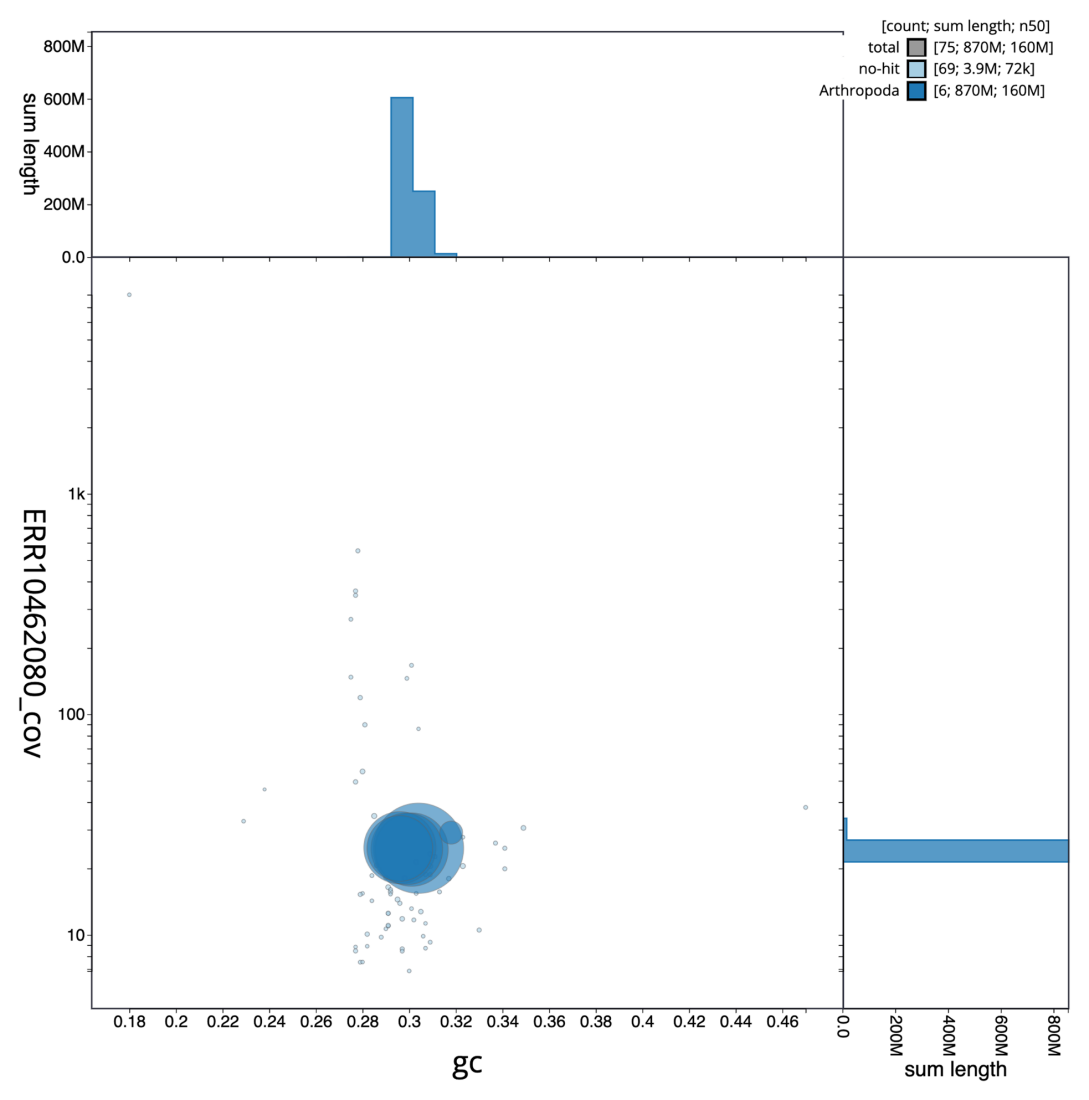
Genome assembly of *Eustalomyia histrio*, idEusHist1.1: BlobToolKit GC-coverage plot. Sequences are coloured by phylum. Circles are sized in proportion to sequence length. Histograms show the distribution of sequence length sum along each axis. An interactive version of this figure is available at https://blobtoolkit.genomehubs.org/view/idEusHist1_1/dataset/idEusHist1_1/blob.

**Figure 4.**
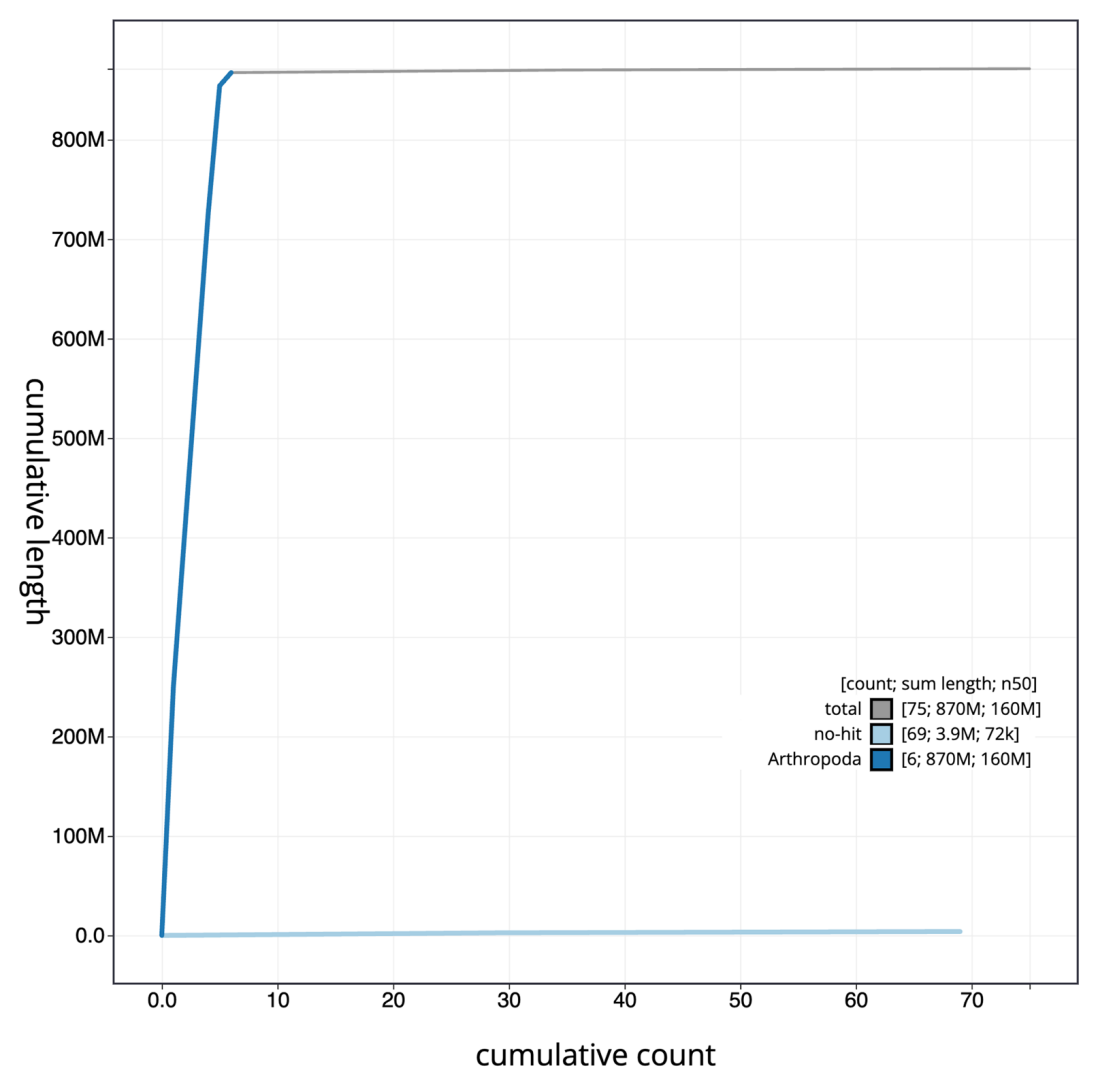
Genome assembly of *Eustalomyia histrio*, idEusHist1.1: BlobToolKit cumulative sequence plot. The grey line shows cumulative length for all sequences. Coloured lines show cumulative lengths of sequences assigned to each phylum using the buscogenes taxrule. An interactive version of this figure is available at https://blobtoolkit.genomehubs.org/view/idEusHist1_1/dataset/idEusHist1_1/cumulative.

**Figure 5.**
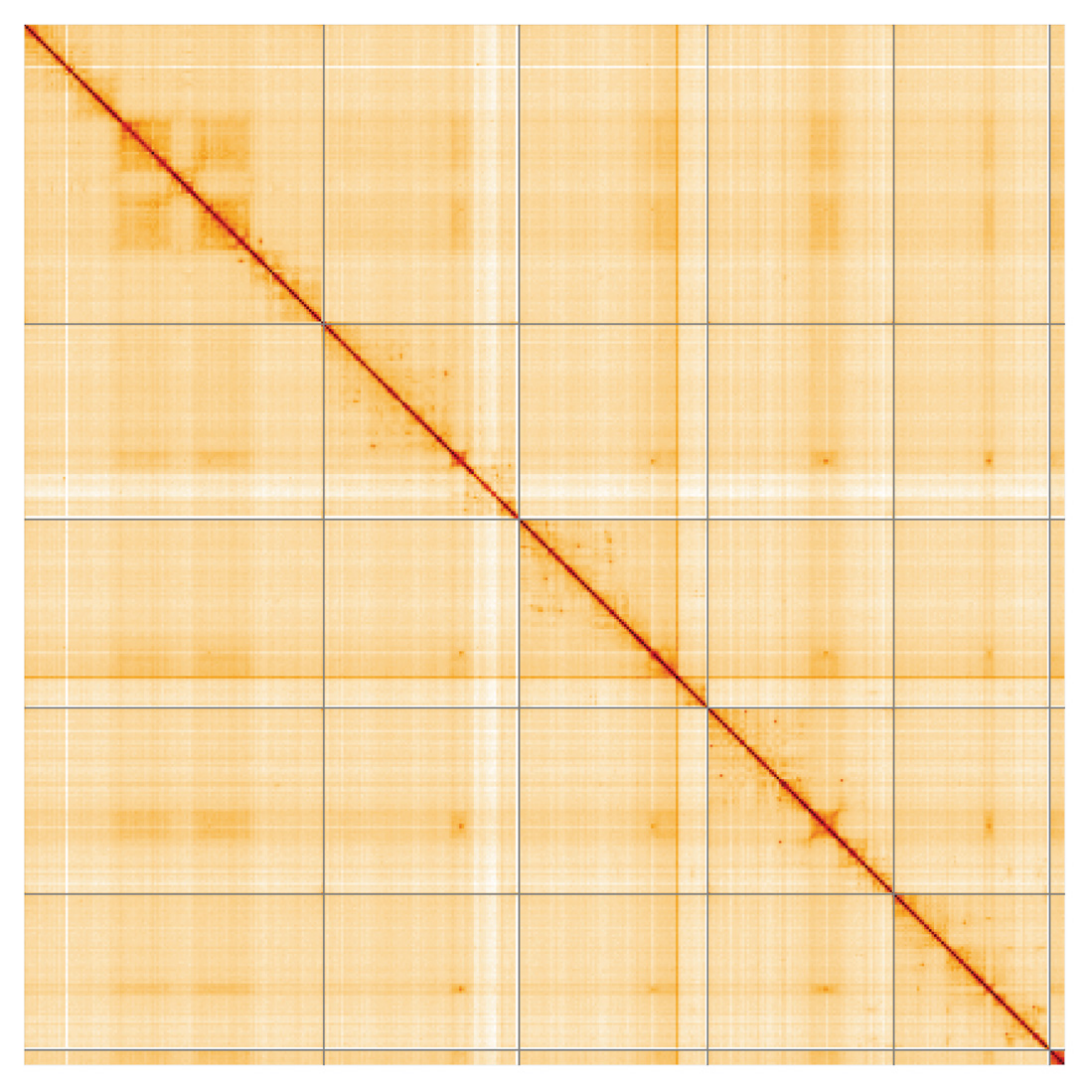
Genome assembly of *Eustalomyia histrio*, idEusHist1.1: Hi-C contact map of the idEusHist1.1 assembly, visualised using HiGlass. Chromosomes are shown in order of size from left to right and top to bottom. An interactive version of this figure may be viewed at https://genome-note-higlass.tol.sanger.ac.uk/l/?d=HPtai6uVRteP0R11f6EGPQ.

The final assembly has a total length of 871.3 Mb in 74 sequence scaffolds with a scaffold N50 of 156.9 Mb ( Table 1). The snailplot in Figure 2 provides a summary of the assembly statistics, while the distribution of assembly scaffolds on GC proportion and coverage is shown in Figure 3. The cumulative assembly plot in Figure 4 shows curves for subsets of scaffolds assigned to different phyla. Most (99.55%) of the assembly sequence was assigned to 6 chromosomal-level scaffolds. Chromosome-scale scaffolds confirmed by the Hi-C data are named in order of size ( Figure 5; Table 2). While not fully phased, the assembly deposited is of one haplotype. Contigs corresponding to the second haplotype have also been deposited. The mitochondrial genome was also assembled and can be found as a contig within the multifasta file of the genome submission.

**Table 1.** Genome data for *Eustalomyia histrio*, idEusHist1.1.

Project accession data		
Assembly identifier	idEusHist1.1	
Species	*Eustalomyia histrio*	
Specimen	idEusHist1	
NCBI taxonomy ID	2881975	
BioProject	PRJEB57280	
BioSample ID	SAMEA110451635	
Isolate information	idEusHist1, whole organism (DNA and Hi-C sequencing)	
Assembly metrics *		*Benchmark*
Consensus quality (QV)	59.5	*≥ 50*
*k*-mer completeness	100.0%	*≥ 95%*
BUSCO **	C:98.3%[S:97.1%,D:1.1%], F:0.5%,M:1.2%,n:3,285	*C ≥ 95%*
Percentage of assembly mapped to chromosomes	99.55%	*≥ 95%*
Sex chromosomes	None	*localised homologous pairs*
Organelles	Mitochondrial genome: 19.42 kb	*complete single alleles*
Raw data accessions		
PacificBiosciences SEQUEL II	ERR10462080	
Hi-C Illumina	ERR10466814	
Genome assembly		
Assembly accession	GCA_949748255.1	
*Accession of alternate haplotype*	GCA_949748445.1	
Span (Mb)	871.3	
Number of contigs	788	
Contig N50 length (Mb)	2.3	
Number of scaffolds	74	
Scaffold N50 length (Mb)	156.9	
Longest scaffold (Mb)	249.79	
Genome annotation		
Number of protein-coding genes	26,785	
Number of gene transcripts	27,436	

* Assembly metric benchmarks are adapted from column VGP-2020 of “Table 1: Proposed standards and metrics for defining genome assembly quality” from Rhie *et al.* (2021). ** BUSCO scores based on the diptera_odb10 BUSCO set using version 5.3.2. C = complete [S = single copy, D = duplicated], F = fragmented, M = missing, n = number of orthologues in comparison. A full set of BUSCO scores is available at https://blobtoolkit.genomehubs.org/view/idEusHist1_1/dataset/idEusHist1_1/busco.

**Table 2.** Chromosomal pseudomolecules in the genome assembly of *Eustalomyia histrio*, idEusHist1.

INSDC accession	Chromosome	Length (Mb)	GC%
OX456523.1	1	249.79	30.5
OX456524.1	2	162.79	30.0
OX456525.1	3	156.86	29.5
OX456526.1	4	154.97	30.0
OX456527.1	5	129.77	29.5
OX456528.1	6	13.26	32.0
OX456529.1	MT	0.02	18.0

The estimated Quality Value (QV) of the final assembly is 59.5 with *k*-mer completeness of 100.0%, and the assembly has a BUSCO v5.3.2 completeness of 98.3% (single = 97.1%, duplicated = 1.1%), using the diptera_odb10 reference set ( *n* = 3,285).

Metadata for specimens, barcode results, spectra estimates, sequencing runs, contaminants and pre-curation assembly statistics are given at https://links.tol.sanger.ac.uk/species/2881975.

## Genome annotation report

The *Eustalomyia histrio* genome assembly (GCA_949748255.1) was annotated using the Ensembl rapid annotation pipeline at the European Bioinformatics Institute (EBI). The resulting annotation includes 27,436 transcribed mRNAs from 26,785 protein-coding genes ( Table 1; https://rapid.ensembl.org/Eustalomyia_histrio_GCA_949748255.1/Info/Index).

## Methods

### Sample acquisition and nucleic acid extraction

A male *Eustalomyia histrio* (specimen ID Ox002194, ToLID idEusHist1) was netted in Wytham Woods, Oxfordshire (biological vice-county Berkshire), UK (latitude 51.77, longitude –1.33) on 2022-05-19. The specimen was collected and identified by Steven Falk (independent researcher), and then snap-frozen on dry ice.

The workflow for high molecular weight (HMW) DNA extraction at the Wellcome Sanger Institute (WSI) includes a sequence of core procedures: sample preparation; sample homogenisation, DNA extraction, fragmentation, and clean-up. In sample preparation, the idEusHist1 sample was weighed and dissected on dry ice ( Jay *et al.*, 2023).

Tissue of the whole was homogenised using a PowerMasher II tissue disruptor ( Denton *et al.*, 2023a), setting aside tissue for Hi-C sequencing. HMW DNA was extracted using the Automated MagAttract v1 protocol ( Sheerin *et al.*, 2023). DNA was sheared into an average fragment size of 12–20 kb in a Megaruptor 3 system with speed setting 30 ( Todorovic *et al.*, 2023). Sheared DNA was purified by solid-phase reversible immobilisation ( Strickland *et al.*, 2023): in brief, the method employs a 1.8X ratio of AMPure PB beads to sample to eliminate shorter fragments and concentrate the DNA. The concentration of the sheared and purified DNA was assessed using a Nanodrop spectrophotometer and Qubit Fluorometer and Qubit dsDNA High Sensitivity Assay kit. Fragment size distribution was evaluated by running the sample on the FemtoPulse system.

Protocols developed by the WSI Tree of Life laboratory are publicly available on protocols.io ( Denton *et al.*, 2023b).

### Sequencing

Pacific Biosciences HiFi circular consensus DNA sequencing libraries were constructed according to the manufacturers’ instructions. DNA sequencing was performed by the Scientific Operations core at the WSI on a Pacific Biosciences SEQUEL II instrument. Hi-C data were also generated from remaining tissue of idEusHist1 using the Arima2 kit and sequenced on the Illumina NovaSeq 6000 instrument.

### Genome assembly, curation and evaluation

Assembly was carried out with Hifiasm ( Cheng *et al.*, 2021) and haplotypic duplication was identified and removed with purge_dups ( Guan *et al.*, 2020). The assembly was then scaffolded with Hi-C data ( Rao *et al.*, 2014) using YaHS ( Zhou *et al.*, 2023). The assembly was checked for contamination and corrected as described previously ( Howe *et al.*, 2021). Manual curation was performed using HiGlass ( Kerpedjiev *et al.*, 2018) and PretextView ( Harry, 2022). The mitochondrial genome was assembled using MitoHiFi ( Uliano-Silva *et al.*, 2023), which runs MitoFinder ( Allio *et al.*, 2020) or MITOS ( Bernt *et al.*, 2013) and uses these annotations to select the final mitochondrial contig and to ensure the general quality of the sequence.

A Hi-C map for the final assembly was produced using bwa-mem2 ( Vasimuddin *et al.*, 2019) in the Cooler file format ( Abdennur & Mirny, 2020). To assess the assembly metrics, the *k*-mer completeness and QV consensus quality values were calculated in Merqury ( Rhie *et al.*, 2020). This work was done using Nextflow ( Di Tommaso *et al.*, 2017) DSL2 pipelines “sanger-tol/readmapping” ( Surana *et al.*, 2023a) and “sanger-tol/genomenote” ( Surana *et al.*, 2023b). The genome was analysed within the BlobToolKit environment ( Challis *et al.*, 2020) and BUSCO scores ( Manni *et al.*, 2021; Simão *et al.*, 2015) were calculated.

Table 3 contains a list of relevant software tool versions and sources.

**Table 3.** Software tools: versions and sources.

Software tool	Version	Source
BlobToolKit	4.2.1	https://github.com/blobtoolkit/blobtoolkit
BUSCO	5.3.2	https://gitlab.com/ezlab/busco
Hifiasm	0.16.1-r375	https://github.com/chhylp123/hifiasm
HiGlass	1.11.6	https://github.com/higlass/higlass
Merqury	MerquryFK	https://github.com/thegenemyers/MERQURY.FK
MitoHiFi	2	https://github.com/marcelauliano/MitoHiFi
PretextView	0.2	https://github.com/wtsi-hpag/PretextView
purge_dups	1.2.3	https://github.com/dfguan/purge_dups
sanger-tol/genomenote	v1.0	https://github.com/sanger-tol/genomenote
sanger-tol/readmapping	1.1.0	https://github.com/sanger-tol/readmapping/tree/1.1.0
YaHS	1.1a.2	https://github.com/c-zhou/yahs

### Genome annotation

The BRAKER2 pipeline ( Brůna *et al.*, 2021) was used in the default protein mode to generate annotation for the *Eustalomyia histrio* assembly (GCA_949748255.1) in Ensembl Rapid Release at the EBI.

### Wellcome Sanger Institute – Legal and Governance

The materials that have contributed to this genome note have been supplied by a Darwin Tree of Life Partner. The submission of materials by a Darwin Tree of Life Partner is subject to the **‘Darwin Tree of Life Project Sampling Code of Practice’**, which can be found in full on the Darwin Tree of Life website here. By agreeing with and signing up to the Sampling Code of Practice, the Darwin Tree of Life Partner agrees they will meet the legal and ethical requirements and standards set out within this document in respect of all samples acquired for, and supplied to, the Darwin Tree of Life Project. 

Further, the Wellcome Sanger Institute employs a process whereby due diligence is carried out proportionate to the nature of the materials themselves, and the circumstances under which they have been/are to be collected and provided for use. The purpose of this is to address and mitigate any potential legal and/or ethical implications of receipt and use of the materials as part of the research project, and to ensure that in doing so we align with best practice wherever possible. The overarching areas of consideration are:

•   Ethical review of provenance and sourcing of the material

•   Legality of collection, transfer and use (national and international) 

Each transfer of samples is further undertaken according to a Research Collaboration Agreement or Material Transfer Agreement entered into by the Darwin Tree of Life Partner, Genome Research Limited (operating as the Wellcome Sanger Institute), and in some circumstances other Darwin Tree of Life collaborators.

## Data Availability

European Nucleotide Archive: *Eustalomyia histrio*. Accession number PRJEB57280; https://identifiers.org/ena.embl/PRJEB57280 ( Wellcome Sanger Institute, 2023). The genome sequence is released openly for reuse. The *Eustalomyia histrio* genome sequencing initiative is part of the Darwin Tree of Life (DToL) project. All raw sequence data and the assembly have been deposited in INSDC databases. Raw data and assembly accession identifiers are reported in Table 1.

## Author information

Members of the University of Oxford and Wytham Woods Genome Acquisition Lab are listed here: https://doi.org/10.5281/zenodo.7125292.

Members of the Darwin Tree of Life Barcoding collective are listed here: https://doi.org/10.5281/zenodo.4893703.

Members of the Wellcome Sanger Institute Tree of Life Management, Samples and Laboratory team are listed here: https://doi.org/10.5281/zenodo.10066175.

Members of Wellcome Sanger Institute Scientific Operations: Sequencing Operations are listed here: https://doi.org/10.5281/zenodo.10043364.

Members of the Wellcome Sanger Institute Tree of Life Core Informatics team are listed here: https://doi.org/10.5281/zenodo.10066637.

Members of the Tree of Life Core Informatics collective are listed here: https://doi.org/10.5281/zenodo.5013541.

Members of the Darwin Tree of Life Consortium are listed here: https://doi.org/10.5281/zenodo.4783558.
