# Supplementary material for: The genome sequence of an anthomyiid fly, Eustalomyia histrio (Zetterstedt, 1838)
Source: Wellcome Open Res. 2024 Apr 24;9:230. [Version 1] doi: 10.12688/wellcomeopenres.21264.1 (PMC11535493; doi:10.12688/wellcomeopenres.21264.1)
Supplement: Peer review history

# Reviewer report: Reviewer response for version 1

**DOI:** 10.21956/wellcomeopenres.23518.r102030

**Published:** 2024-11-04

**Authors:** Kah-Ooi Chua

**Affiliations:** 1 Universiti Malaya, Kuala Lumpur, Federal Territory of Kuala Lumpur, Malaysia

The authors report a high quality whole genome sequence of *Eustalomyia histrio*, an Anthomyiid fly. There are only a few minor comments and suggestions.

 In the introduction, it would be great if some info about the ecological role and economic impact of this fly species is provided, in addition to its dietary habits (nectar and pollen feeding). It will help the readers understand if this species is a pest, a pollinator, or it is playing other roles in its ecosystem.

 It was mentioned that “A total of 24-fold coverage in Pacific Biosciences single-molecule HiFi long reads was generated.” But the coverage of the final genome assembly that included Illumina data was not provided.

 In the Methods section under Sample acquisition and nucleic acid extraction, correct the ; to , after sample preparation;

 Under Sequencing section, more information could be provided for Illumina sequencing setup in terms of read length and cycle setting (e.g. 2x150 bp or other setting). This information can help readers understand the data resolution when they compare it to other dataset.

 Suggest to amend the label for X-axis in Figure 4 to cumulative scaffold count.

 A scale bar is needed for the fly specimen in Figure 1 to help readers understand the size of the fly species.

 The mitochondrial genome assembled had a length of 19.42 kb, which is longer than average length of animal mitochondrial genome length of 16.5 kb. Perhaps, the authors can look into the gene contents of the mitochondrial genome to see if there are any gene duplication or non-coding regions, and discuss if this is normal for flies in the same taxon.

Are sufficient details of methods and materials provided to allow replication by others?

Yes

Is the rationale for creating the dataset(s) clearly described?

Yes

Are the datasets clearly presented in a useable and accessible format?

Yes

Are the protocols appropriate and is the work technically sound?

Yes

Reviewer Expertise:

Insect organelle genomics

I confirm that I have read this submission and believe that I have an appropriate level of expertise to confirm that it is of an acceptable scientific standard.

# Reviewer report: Reviewer response for version 1

**DOI:** 10.21956/wellcomeopenres.23518.r99490

**Published:** 2024-09-22

**Authors:** Ruiqi Li

**Affiliations:** 1 University of Colorado Boulder, Boulder, Colorado, USA

Falk et al. present a high-quality genome of the anthomyiid fly, *Eustalomyia histrio* (Zetterstedt, 1838). While the methods appear rigorous, I have a few minor suggestions for improvement.

 I like how the authors introduce the taxonomy, natural history, distribution of the species. Great work!

 Is there any other notable biological aspect of *E. histrio* that could be explored using the genome data? You might consider including examples of questions that the genome could help address, see some examples from Li et al. 2024.

 Could you please add a scale to the specimen photo?

Are sufficient details of methods and materials provided to allow replication by others?

Yes

Is the rationale for creating the dataset(s) clearly described?

Yes

Are the datasets clearly presented in a useable and accessible format?

Yes

Are the protocols appropriate and is the work technically sound?

Yes

Reviewer Expertise:

Genomics

I confirm that I have read this submission and believe that I have an appropriate level of expertise to confirm that it is of an acceptable scientific standard.

## References

[1] : The genome sequence of a heart cockle, Fragum fragum (Linnaeus, 1758). *Wellcome Open Res* .2024;9: 10.12688/wellcomeopenres.21134.1 129 10.12688/wellcomeopenres.21134.1 38989474 PMC11234083

[2] : The genome sequence of the giant clam, Tridacna crocea (Lamarck, 1819). *Wellcome Open Research* .2023;8: 10.12688/wellcomeopenres.20580.1 10.12688/wellcomeopenres.20580.1

[3] : The genome sequence of the giant clam, Tridacna gigas (Linnaeus, 1758). *Wellcome Open Res* .2024;9: 10.12688/wellcomeopenres.21136.1 145 10.12688/wellcomeopenres.21136.1 38800516 PMC11116938
